# Is Ockham’s razor losing its edge? New perspectives on the principle of model parsimony

**DOI:** 10.1073/pnas.2401230121

**Published:** 2025-01-27

**Authors:** Marina Dubova, Suyog Chandramouli, Gerd Gigerenzer, Peter Grünwald, William Holmes, Tania Lombrozo, Marco Marelli, Sebastian Musslick, Bruno Nicenboim, Lauren N. Ross, Richard Shiffrin, Martha White, Eric-Jan Wagenmakers, Paul-Christian Bürkner, Sabina J. Sloman

**Affiliations:** ^a^Cognitive Science Program, Indiana University, Bloomington, IN 47405; ^b^Santa Fe Institute, Bloomington, NM 87501; ^c^Department of Information and Communications Engineering, Aalto University, Espoo FI-00076, Finland; ^d^Department of Computing Science, University of Alberta, Edmonton, AB T6G1T6, Canada; ^e^Max Planck Institute for Human Development, Berlin 14195, Germany; ^f^Centrum Wiskunde & Informatica, Amsterdam 1098 XG, The Netherlands; ^g^Department of Statistics, Mathematical Institute, Leiden University, Leiden 2311 EZ, The Netherlands; ^h^Department of Psychology, Princeton University, Princeton, NJ 08544; ^i^Department of Psychology, University of Milano-Bicocca, Milan 20162, Italy; ^j^Institute of Cognitive Science, Osnabrück University, Osnabrück 49090, Germany; ^k^Department of Cognitive, Linguistic, and Psychological Sciences, Brown University, Providence, RI 02912; ^l^Department of Cognitive Science and Artificial Intelligence, Tilburg University, Tilburg 5037 AB, The Netherlands; ^m^Department of Logic and Philosophy of Science, University of California, Irvine, CA 92697; ^n^Psychological and Brain Sciences Department, Indiana University, Bloomington, IN 47405; ^o^Psychological Methods, Psychology Research Institute, University of Amsterdam, Amsterdam 1018 WT, The Netherlands; ^p^Department of Statistics, Technical University Dortmund University, Dortmund 44227, Germany; ^q^Department of Computer Science, University of Manchester, Manchester M13 9PL, United Kingdom

**Keywords:** scientific modeling, parsimony, complexity, Ockham’s razor

## Abstract

The preference for simple explanations, known as the parsimony principle, has long guided the development of scientific theories, hypotheses, and models. Yet recent years have seen a number of successes in employing highly complex models for scientific inquiry (e.g., for 3D protein folding or climate forecasting). In this paper, we reexamine the parsimony principle in light of these scientific and technological advancements. We review recent developments, including the surprising benefits of modeling with more parameters than data, the increasing appreciation of the context-sensitivity of data and misspecification of scientific models, and the development of new modeling tools. By integrating these insights, we reassess the utility of parsimony as a proxy for desirable model traits, such as predictive accuracy, interpretability, effectiveness in guiding new research, and resource efficiency. We conclude that more complex models are sometimes essential for scientific progress, and discuss the ways in which parsimony and complexity can play complementary roles in scientific modeling practice.

“*Plurality should not be posited without necessity”*

–*Ockham, 13xx/1986*

Imagine you are assessing the effectiveness of a new, untested drug. Should you start with the parsimonious assumption that the treatment has no beneficial effect until proven otherwise? Now consider aspirin, a drug known to relieve pain. Given its proven effects on human physiology, should you still assume that it has no side effects? For aspirin, but not the untested drug, it might be more reasonable to make the less parsimonious assumption that the drug has multiple side effects (1). Now, imagine you are choosing an approach to study human language acquisition. Chomskyan linguistics aims to explain the richness and expressivity of human languages by positing a parsimonious set of universal grammatical rules (2). Conversely, modern large language models (LLMs) are highly complex, learning from vast datasets without strong priors over possible linguistic structures. These models generate coherent, human-like text, and despite their complexity, offer scientific insights that traditional theories do not (3).

These examples highlight the nuanced role of parsimony in modern scientific practice. This paper asks: When are more parsimonious models beneficial? What are they beneficial for? And what is model parsimony in the first place? We first distinguish between two competing understandings of “parsimony” in scientific modeling, one based on a model’s flexibility in fitting observed data and the other based on the model’s number of meaningful components (e.g., causes, mechanisms, or variables) (Section 2, Box 1). Then, we review the ways in which these two forms of parsimony do and do not align with generally desirable properties of scientific models, such as predictive accuracy, interpretability, and others. We focus on new tools and results that reinforce or challenge traditionally assumed relationships between parsimony and these desirable properties of models (Section 3). We conclude that parsimony is not a universal guide to scientific progress; rather, parsimony and complexity should be employed as complementary principles in scientific modeling (Section 4). Rather than substituting existing in-depth resources, this paper serves as an entry point, inspired by recent counterintuitive findings that challenge common views on parsimony in science, and aims to inspire further research into its evolving role.

Box 1. Glossary.

**Scientific model**: Formal representation of a target phenomenon. Some (process or mechanistic) models reflect generative processes, while others simply describe the data.

**Parsimony by components** (Section 2.1): The principle of preferring a model that contains a smaller number of meaningful components (e.g., causes or mechanisms).

**Parsimony by constraints** (Section 2.1): The principle of preferring a model that is less flexible, or could capture a smaller number of distinct data patterns.

**Model fitting/training**: The process of identifying the instance of a model that best matches the data (e.g., identifying the best-fitting parameter values or structure).

**Model misspecification**: When a model cannot capture the data-generating process, regardless of the training data and fitting procedure.

**Bias**: Error attributable to systematic factors, like structural limitations of a model (e.g., misspecification), properties of the training data, or the fitting procedure (Fig. 1).

**Fig. 1.**
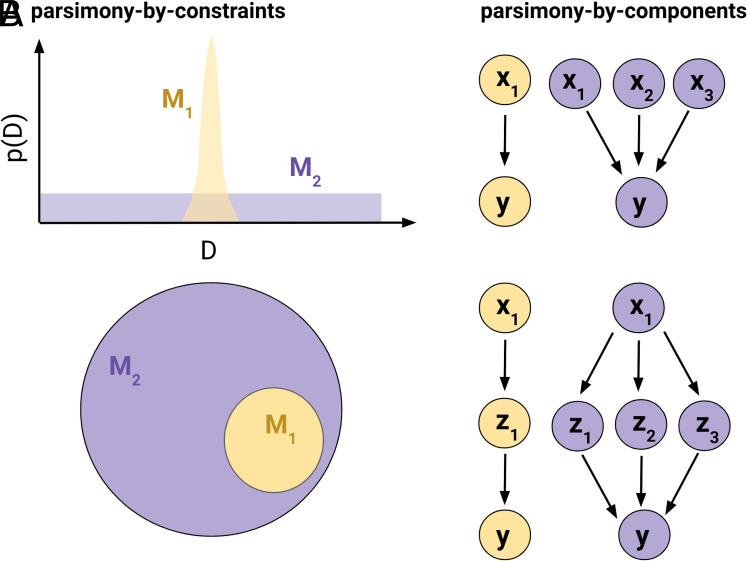
Illustration of different forms of parsimony. (*A*) Parsimony by constraints. *Upper*: A more parsimonious model (yellow) assigns a high probability to only a narrow range of events, while a more complex model (purple) widely spreads its predictions. *Lower*: A more parsimonious model (yellow) captures a subspace of phenomena that a more complex model (purple) can accommodate. (*B*) Parsimony by components. *Upper*: A parsimonious model (yellow) works with fewer input variables than a more complex model (purple). *Lower*: A parsimonious model (yellow) postulates fewer latent variables/causes than a more complex model (purple).

**Variance**: Error attributable to a model’s sensitivity to noise in the training data. Models with high variance capture noise instead of regularities, or overfit, the training data (Fig. 1).

**Regularization**: Techniques used to reduce a model’s variance by penalizing its complexity.

## 1. Is Parsimony Used in Modern Scientific Practice?

Considerations of parsimony lie at the core of our everyday reasoning. When laypeople assess explanations, they often prefer explanations that appeal to fewer causes (4, 5) or that are less flexible in accommodating the data (Fig. 1*A*; 6). Similarly, scientists prefer to explain a collection of findings by appealing to one unifying mechanism rather than several distinct mechanisms or factors. This need for parsimonious accounts has been linked to limitations of human cognition that affect our ability to generate, understand, use, and communicate explanations (7, 8).

Parsimony also has a key place in the development of scientific models, playing formal and informal roles in how scientists identify, construct, and assess their models. Across scientific domains, developing a more complex model often needs to be justified by stronger evidence, whereas coming up with a more parsimonious model is viewed as a scientific contribution in itself (9–11). When designing a new model, scientists are often trained to start with a simple candidate model and to iteratively scale it up until it captures the essential aspects of the data (12, 13). Parsimony plays a central role in formal model selection: For example, popular measures for selecting between models or hypotheses [e.g., AIC, BIC, Bayes Factor (12, 14–16)] attempt to strike an optimal balance between goodness of fit and parsimony (17, 18).^*^

The historical preference for parsimonious models stands in contrast with recent developments in science and technology, which have demonstrated that extremely complex models can facilitate scientific progress by, e.g., predicting the 3D structures of proteins (21), improving climate forecasts (22), and enhancing scientific understanding of the mechanisms of language acquisition (3). As a result, some disciplines—including physics (23), systems biology (24), and medicine (25)—are becoming less concerned with parsimony. Other fields such as finance, engineering, and computer science often rely on complex models, sometimes overlooking simpler alternatives that can lead to more robust and accurate results (26–31). With more complex models being increasingly adopted in scientific disciplines, there is a need to reexamine the role that parsimony plays in science.

While our intuitive preference for parsimony has shaped scientific practice, this preference is neither universal nor universally beneficial. A preference for parsimony has not been obviated by scientific advances; rather, its usefulness depends on the modeling context and goals (Section 3). Recognizing that parsimony is just one of many tools available to scientists is crucial for advancing our ability to learn about the world (Section 4).

## 2. What is Model Parsimony?

In this section, we articulate two ways in which model parsimony has been conceptualized: on the basis of either a model’s flexibility or the number of meaningful components it consists of. These competing understandings of parsimony have been used interchangeably to justify parsimony as a criterion for scientific modeling. We then discuss the process of specifying a parsimonious model, which requires a set of judgments on the part of the scientist. This background will set the stage for our main discussion of the utility of parsimonious models across scientific contexts in Section 3.

### 2.1. Two Forms of Parsimony.

Model parsimony is a multidimensional concept that has been thought about in many, sometimes contradictory, ways. Many notions of parsimony can be organized into two broad categories.^†^ These categories will be essential for understanding the arguments for and against the utility of parsimonious models for different modeling goals in Section 3.

#### 2.1.1. Parsimony by constraints.

(Fig. 1*A*) appeals to a model’s limited flexibility, or lack of capacity to accommodate different potential patterns in the data.^‡^ Models that are parsimonious in this way anticipate specific empirical outcomes with greater confidence. To illustrate, consider the model predicting the effect of an untested drug. A parsimonious model might predict that the drug has no effect. This prediction is specific and narrow-it only allows for outcomes in which the drug has no effect at all. On the other hand, a less parsimonious model might predict that the drug could have any effect, whether positive or negative, and of any magnitude. This latter model is more flexible and can accommodate a wider range of possible outcomes, and thus, is less parsimonious by constraints. Bayesian instantiations of parsimony align with this intuition of parsimony by constraints (32, 33). To enforce parsimony by constraints, scientists typically select models with fewer parameters or effective parameters (34, 35), less expressive functional forms (36), more precise prior distributions (32, 37), shorter description length (38–40), lower rank (41), or other criteria.

#### 2.1.2. Parsimony by components.

(Fig. 1*B*) defines the complexity of a model as the number of meaningful components it has (4, 38, 42, 43). These components can include types or instances of variables, independent and root causes, or distinct processes represented in the model. To illustrate, consider our example of modeling human language. A parsimonious-by-components model would aim to explain the richness of human languages with a minimal set of grammatical rules. For example, Chomsky’s Theory of Universal Grammar proposes that a small number of fundamental rules can account for the vast diversity of languages spoken around the world. In contrast, a less parsimonious model might employ a larger set of rules tailored to different languages. While this model might explain the structure of different languages more precisely, it is more complex by components because it postulates more rules.

While distinct, these two forms of parsimony are interconnected (Box 2). Moreover, assessing model parsimony in practice requires many nuanced choices (Box 3). Some of the ensuing discussion in Section 3 will apply to models that are both parsimonious by constraints and by components, in which cases we will not explicitly distinguish between the two forms. In other parts, we highlight where the two notions of parsimony diverge, showing that often only one—or neither—aligns with certain desirable model properties.^§^

Box 2. (in-depth). Alignment of two forms of parsimony.

Parsimony by components and by constraints align when model components can adjust based on observed data. For example, adding input variables (components) in multiple regression expands the set of input–output relationships consistent with the model. The two forms also align when there is uncertainty about the model components because this uncertainty allows for a wider range of possible model behaviors. For example, state-of-the-art climate models incorporate many structural hypotheses about which there is substantial disagreement or uncertainty within the scientific community. Because of this uncertainty, a given climate model can be consistent with many outcomes (44). Misalignment between the two forms of parsimony occurs when model components are neither adjustable nor uncertain. For example, quantum electrodynamics posits many theoretical components but makes extremely precise predictions, and is in that sense inflexible (45).

### 2.2. Specifying a Parsimonious Model.

Specifying parsimonious models requires that the modeler make a series of judgments as to what components to include or constraints to impose. One might impose a linear relationship between the model’s inputs and its predictions, give the model access to only a few key variables to predict the outcome, or assign more precise prior distributions to the model parameters. These judgments can incorporate a variety of considerations, which include specific modeling goals and prior domain knowledge about the structure of the target phenomena. Well-specified models resemble the target phenomena.

Box 3. (in depth). Using parsimony.

Assessing a model’s parsimony introduces many nontrivial issues. For example, it is not clear whether parsimony should be a property of the model itself or of the model relative to the data it describes (e.g., number of parameters is typically a property of the model independent of the data, while the effective number of parameters is a relative property). Moreover, depending on the context, one may want to evaluate the parsimony of a class of models or of a specific model instance (i.e., a model with its structure and parameter values fixed). A related concern is when parsimony should be estimated—before or after the model is fitted to data. Finally, when counting components or constraints of a model, it is often unclear what exactly counts as a component or constraint. For example, one might have to decide how abstract the components could be or whether nodes, root causes, or variables constitute independent components.

Another significant challenge involves choosing a way to integrate parsimony into the scientific modeling process. In many formal procedures, parsimony trades off with goodness of fit (e.g., in AIC or BIC) or it is used as a tie-breaker when choosing between two models that otherwise perform equally well. More informally, parsimony is often a key consideration when scientists choose a model to start with; for example, they might prefer to start with a minimal causal model that only contains one key variable, and only expand this model if there is sufficient evidence that additional variables are at play. In real-world applications, the evaluation and value of parsimony depend on how it is defined, instantiated, and incorporated into the scientific modeling process.

For many purposes, however, models are deliberate oversimplifications (46), i.e., the modeler is aware that there is a resemblance gap between the target phenomenon and the model. When this gap is present (whether deliberately or not), the model is misspecified. All else equal, models that are less parsimonious by constraints can extract a larger number of patterns from data, and so generally run a lower risk of misspecification.

## 3. Are Parsimonious Models More Useful Models?

“*All models are wrong, some are useful”*

–*Box, G., 1976*

“*Some models are useful, but how do we know which*

*ones?”*

–*Bürkner, Scholz & Radev, 2023 (47)*

In this section, we discuss the reasons why parsimonious models should or should not be preferred in specific situations. We assess model parsimony as a tool for achieving specific desirable model characteristics. In particular, we discuss the degree to which the parsimony principle guides—and *mis*guides—scientists in pursuit of models that are more accurate in their predictions (Section 3.1), interpretable (Section 3.2), useful in guiding new research (Section 3.3), resource efficient (Section 3.4), suitable for working with limited data (Section 3.5), or aligned with domain-specific assumptions about target phenomena (Section 3.6). These model properties are associated with different scientific goals, which include understanding phenomena, predicting future events, controlling systems, and communicating scientific knowledge to different audiences, from children to scientific communities.^¶^

We note that much of the discussion in this section is technical and particularly relevant for those who employ models in their work. For readers seeking a more introductory and accessible overview of parsimony, we recommend (43) and (42).

### 3.1. Predictive Accuracy.

Models are often used to inform our expectations of new events based on past observations. In this section, we discuss the way parsimony affects the accuracy of a model’s predictions. Unless otherwise specified, here we use the word *parsimony* in the sense of parsimony by constraints.

Parsimony can both help and hurt a model’s ability to make accurate predictions. Parsimony helps when models are simplified based on reliable assumptions, leading to robust predictions even from noisy data. However, parsimony hurts when these assumptions are incorrect, making the model unable to capture the structure in the data. Moreover, while the expressivity of complex models was traditionally thought to be detrimental for their robustness, recent research shows that complex models can also exhibit high robustness to noisy data.

#### 3.1.1. Simplifying assumptions.

Specifying a parsimonious model necessitates positing assumptions to inform the simplifying constraints (Section 2.2). Well-specified models efficiently learn from data and make accurate predictions in new situations (48). Domain-specific knowledge often informs constraints that enhance predictive accuracy, such as assuming most variables do not affect the outcome, that variables are linearly related, or that only recent history matters for future predictions. For example, when assessing the effectiveness of a new, untested drug, starting with the assumption that the drug has no effect until proven otherwise can lead to more reliable predictions if most drugs indeed have no effect. Similarly, in many cases of predicting human behavior in the real world, e.g., in court decisions on recidivism (49) or when predicting the future of fragile families (50), there is little evidence that complex models are superior to parsimonious ones that rely only on a few of the most important variables (51).

What if one lacks well-informed assumptions to simplify the model? Imposing constraints on a model restricts the number of patterns it can account for. When constraints are based on misinformed assumptions, the parsimonious model becomes misspecified, and is unable to capture the structure in the data (32, 52). For example, given aspirin’s known effects on human physiology, models that anticipate that it also has multiple side effects might yield more accurate predictions than those assuming no effects (1). Misspecified models learn less efficiently (53, 54) and can even “hallucinate” patterns that are not present in the data. For example, parsimonious models used to analyze brain activity can erroneously infer the presence of oscillatory components when brain activity slowly changes in space and time (55). In quantitative genetics, imposing constraints on genetic covariance matrices can lead to biased estimates, with models often learning the wrong subset of principal components (56). More complex (less constrained) models are better equipped to account for the data patterns they encounter.

In many disciplines, unduly constrained modeling is linked to low prediction accuracy. Parsimonious-by-components models that assume that only a few factors affect the outcome can fail to make accurate predictions in contexts where these excluded factors do matter. For example, many models in the psychological and social sciences abstract away from details of the populations, times, locations, and other contextual aspects of the data. These details are, however, considered as key variables that affect psychological outcomes (57–59). Thus, some of the constraints used to simplify scientific models are not aligned with domain-specific knowledge that could improve a model’s predictions.

#### 3.1.2. Robustness.

Conventional wisdom suggests that models with many parameters, which are complex by both components and constraints, are prone to overfitting: The model’s parameters are fit so closely to the observed data that they capture noise instead of stable patterns. These models often capture the observed data better (decrease bias; Section 3.1.1), but slight perturbations of the observed data lead them to make wildly different predictions (increase variance). This idea is captured by the bias-variance trade-off (52).

*Explicit regularization* techniques are often applied to improve robustness, encouraging the fitted models to be more parsimonious. Some forms of explicit regularization promote sparsity, enforcing parsimony by components by limiting the number of parameters. Others favor instances of the model that balance a close fit to the data with small parameter values.

**Fig. 2.**
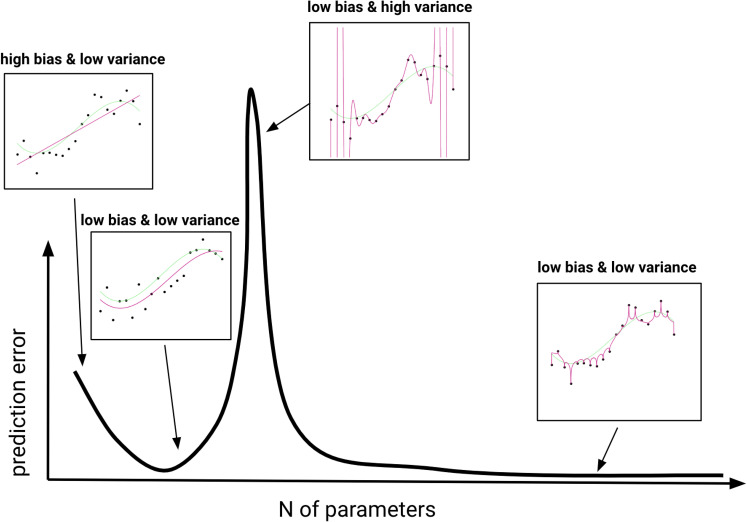
Double descent of prediction error. Degree-one, degree-three, degree-twenty, and degree-one-thousand polynomial regression fits (magenta; from *Left* to *Right*) to data generated from a degree-three polynomial function (green). Low prediction error is achieved by both degree-three and degree-one-thousand models. Figure adapted from ref. 67.

Interestingly, recent findings show that robustness can be achieved without explicit regularization, i.e., without an expressed preference for model parsimony. For example, models with many more parameters than data points can achieve both low variance and low bias (60–63). Fig. 2 illustrates this phenomenon, known as double descent, where increasing number of parameters first raises and then lowers prediction error. As the number of parameters continues to grow, bias steadily decreases, while variance follows a bell-shaped curve, enabling models with an excess of parameters to achieve smooth and robust fits (64–66). Open questions remain, such as how well these apparently complex models generalize to entirely new settings.

The work on the double descent phenomenon suggests that although increasing the number of parameters increases the number of components and reduces the constraints imposed by the model structure, the process of model fitting is nevertheless guided toward model instances that generalize well. This may be due to the behavior of commonly used model selection and model-fitting algorithms (65, 68). These implicit constraints on how the process of model fitting navigates the space of possible model instances are known as *implicit regularization*. This finding has prompted a line of work exploring new ways to assess parsimony for models with an excessive number of parameters (69, 70).

Double descent shows that i) the constraints placed on a model can come from a variety of, sometimes surprising, places that do not reflect the expressed preference of the modeler, and that ii) explicitly favoring more parsimonious models is not the only path to robust predictions; often, models that are extremely expressive and unconstrained in some senses can discover more robust solutions.

### 3.2. Interpretability.

Many scientists use models to better understand phenomena of interest. Here, we discuss the relationship between a model’s parsimony and interpretability. We consider two forms of interpretability: *Intrinsic interpretability* refers to an understanding of the inner workings of the model itself, while *external interpretability* refers to insights about the real-world phenomena gained from using the model. In this section, we use the word “parsimony” in the sense of parsimony by components.

Parsimony helps intrinsic interpretability when it aligns with human cognitive limitations, making models easier to understand and use. However, this relationship is not universal given the subjective nature of intrinsic interpretability and new techniques that make complex models more understandable (Section 3.2.1). Conversely, parsimony may harm external interpretability when it leads to model misspecification, resulting in biased representations of the world—in such cases, an understandable model fails to communicate the structure of the world and, therefore, fails to serve as a powerful explanatory tool for a scientist (Section 3.2.2).

#### 3.2.1. Intrinsic interpretability.

Let us first discuss intrinsic model interpretability defined as humans gaining a sense of understanding of what the model is doing.^#^ In many real-world decision-making scenarios, it is important that humans can use models in a transparent and reliable way. When a model has few components it might be easier for people to 1) anticipate the qualitative nature and scope of the patterns the model predicts, and 2) understand the effects that model components have on the model’s predictions. Models with many components can easily become unintelligible (74, 75). Parsimonious models, when they are more intrinsically interpretable, are more usable in real-world scenarios. For example, parsimonious tree models have helped doctors when prioritizing victims at the site of an accident or military personnel when making life-and-death decisions at military checkpoints (76).

However, parsimony is not a universal guide to intrinsic interpretability. Some parsimonious models are very difficult to interpret, whereas some complex models are very intrinsically interpretable. For example, interpreting nonlinear models can be very difficult. A nonlinear model with just a single parameter can produce patterns of arbitrary complexity, from a linear trend to the contour of an elephant, making it extremely challenging to interpret that single parameter (77). Neural network models with only two neurons can produce a great variety of behaviors, with intensive study devoted to understanding the scope of these behaviors (78). On the other hand, specific structural choices can make complex models more interpretable than their more parsimonious counterparts. For example, a *sparse* model with many parameters, only a few of which are sensitive to a given model’s input, might be seen as less parsimonious by components than a dense model with few parameters, each of which responds to a variety of inputs. However, the sparse model might actually be more interpretable, since the correspondence between the inputs and parameters can be more easily inferred. These examples highlight that parsimony is not a reliable proxy for intrinsic model interpretability.

Another reason why intrinsic model interpretability can come apart from parsimony is that model users can differ in how they understand the model. Some models can include many low-level components, but still be very interpretable at a more abstract level. Intrinsic interpretability often hinges on a scientist’s prior knowledge about the domain and their ability to break down the model into modules and/or a hierarchy (79–81). For example, O’Reilly and Frank (2006)’s (82) computational model of working memory includes thousands of neurons whose learning dynamics are determined by the areas these neurons are located in. While difficult to interpret at the level of individual neurons and their interactions, this model offers insights when abstracting from individual neurons and considering brain areas. Parsimony in either form fails to account for the differences in expertise and prior knowledge that affect individuals’ ability to interpret scientific models.

The relationship between parsimony and intrinsic interpretability is further complicated by new methods developed to understand the workings of complex models. Methods for interpreting complex models include “explainable AI” techniques, which peer into the black box of complex models (83, 84). Additionally, scientists employ cognitive experiments, which treat models as participants to systematically assess their responses to stimuli, and ablation studies, which systematically remove parts of a model to assess their impact on performance, to gain insights into complex models (85, 86).

#### 3.2.2. External interpretability: Relating the model back to the world.

Producers and consumers of models are typically interested not only in understanding how the model itself is operating, but in using the model as a tool to explain the world. This involves assessing whether the model’s components and their estimates accurately reflect relevant aspects of the target phenomenon. For example, when interpreting the estimated effect of age on susceptibility to a certain drug in a regression model, one might be interested in the estimated age parameter to the extent that it characterizes the relationship between age and susceptibility in the real world. Thus, external interpretability ensures that the components of the model provide accurate and relevant insights into the phenomena being studied (87).

As discussed in Sections 2.2 and 3.1.1, the ability of a parsimonious model to faithfully represent the target phenomenon depends on the quality of the assumptions used in the specification of the model. Given the presence of or potential for model misspecification, the relationships between the components of the model are distinct from the relationships between the corresponding aspects of the world, and so more parsimonious models can exhibit lower external interpretability. The components of the model then offer little in terms of reflecting the structure of the target phenomenon or anticipating the effect of changes in the world (88). For example, Wagenmakers et al. (89) provide a demonstration of how a relationship between two variables (LEGO price and the minimal age printed on the box) can be estimated as positive (LEGO boxes for older kids are more expensive), negative (LEGO boxes for younger kids are more expensive), or null, depending on whether important mediators (e.g., the number and weight of the LEGO pieces) are included in the model (see refs. 90 and 91 for other examples). Similarly, when estimating the effect of an untested drug, omitting key mediators can lead to qualitatively different conclusions about its effectiveness. In this way, making a model more complex is often required to use it to faithfully represent and interpret the world.

### 3.3. Effectiveness in Guiding New Research.

Models help scientists conduct new research, for example, by helping them design better experiments or choose more useful questions to be addressed in future studies. In this section, we explore parsimony as a heuristic for building models that are successful at guiding scientists to conduct better research.

#### 3.3.1. Guiding scientific reasoning.

Parsimonious models can serve as helpful reasoning tools. More parsimonious models invite new research more readily when they are easier for scientists to intuit, adapt to new settings, or anticipate empirical implications of (Section 3.2.1). To the extent that the specific form of parsimony conforms to the constraints of scientific reasoning and communication,^‖^ parsimonious models can serve as helpful “intuition pumps” (92). Moreover, the process of constructing parsimonious models might lead a scientist to conceptualize new features or dimensions, putting them in a better position to ask new research questions (71, 93–95). Thus, parsimony enhances scientific reasoning when it effectively interacts with the constraints of human cognition.

#### 3.3.2. Guiding research paradigms.

Relying on parsimonious models to guide new research results in biases in the scientific process; whether or not these biases are desirable is a matter of context and debate. For example, models that are parsimonious by constraints may result in science progressing as a series of sequential hypothesis tests (96, 97).

Historically, parsimonious-by-components models have been associated with generality, suggesting that models with fewer domain-specific details can inspire research across diverse fields (98), although this relationship has recently been challenged (99). Conversely, using parsimonious models has also been linked to fragmented and focused efforts to study toy problems in well-contained scenarios (100–102). For example, attention, perception, memory, and categorization are all pursued as different subfields in cognitive psychology, each of which uses parsimonious models to explain behavior in narrow ranges of well-controlled environments. Setting up new studies to test these models often results in reusing the narrow paradigms that these models are designed to capture. To illustrate, scientists comparing decision models that assume a small number of attributes to be relevant often rely on stylized experimental paradigms where participants compare options that differ only in these attributes. Alternative, integrative modeling approaches instead aim to develop mechanistic accounts that capture phenomena in a wide range of scenarios (e.g., human behavior across tasks), and typically lead scientists toward more complex, context-sensitive models, such as cognitive architectures, convolutional neural networks, and LLMs (102–105). Parsimonious models can help or harm the scientific process depending on whether the biases they introduce are aligned with scientific goals.

#### 3.3.3. Guiding scientific experimentation.

To the extent that they are more likely to be misspecified (Sections 2.2 and 3.1.1), parsimonious models can harm scientific experimentation by pointing scientists in wrong directions. When we rely on misspecified models to guide new research, we expose the scientific process to bias twice: First, when drawing inferences from the misspecified model, and again when making decisions about which research to pursue next (106). Misspecified models risk guiding future research in unproductive directions (107–109), in ways that can be difficult to detect (110). Dubova et al. (110) examine the success of scientists in coming up with a good model of the world by conducting experiments that are either informed by their current model of the world or are simply chosen at random. In this computational study, the community of scientists is set up to conduct targeted experiments that either aim to distinguish between competing models of the world, falsify or confirm them, or to use the scientists’ knowledge in some other way. Importantly, the scientists’ models of the world were misspecified and were subject to incremental improvement. Across all the tested contexts, conducting experiments at random resulted in learning more informative and predictive models of the world than conducting experiments informed by such misspecified accounts (see also ref. 111). Parsimonious models that are deliberately simplified approximations of the world are often misspecified, and so could similarly misguide scientific experimentation.

### 3.4. Resource Efficiency.

Models that require fewer resources (e.g., memory and time) for training and use can be more easily developed, shared, and used by researchers without access to expensive hardware. Parsimony has traditionally helped scientists develop resource-efficient models. However, recent advancements reveal that complex models can effectively approximate less resource-efficient parsimonious models, indicating that strictly adhering to parsimony may harm resource efficiency. Below, we examine resource efficiency in both model *training* and *usage* contexts.

#### 3.4.1. Training.

Parsimonious models can be easier to train. For example, the time it takes to infer the best model can grow polynomially or even exponentially with the number of flexible parameters or data points. Moreover, linear algebra and regularization operations often require more energy and memory as the number of parameters increases. As a consequence, training large neural network models (e.g., LLMs) has been shown to impose a large environmental cost (112, 113). Moreover, parsimonious models with well-studied parametric forms often admit analytical solutions, which can save substantial resources for optimization. For example, there are analytical solutions that enable fast fitting of drift diffusion models (114), as compared to more complex alternatives that may better explain the data but cannot be as widely used because of the optimization challenges [e.g., leaky competing accumulator: (115)]. Therefore, parsimony can guide the development of models that are easier to train.

Recent work has blurred the association between a model’s parsimony and the resources it needs to be trained. Sometimes, making a model less parsimonious (e.g., by increasing its number of parameters) can make it more computationally tractable. For example, training models with more parameters than data can be faster and more successful than training models with fewer flexible parameters (116–119). Finally, there are now complex machine learning models being used in place of more parsimonious models that are too slow or even intractable to train (120, 121). For example, Sukys et al. (122) developed a neural network model to predict the solutions for the Chemical Master Equation across a range of parameters, thus overcoming the need to use expensive simulation and approximation techniques to solve the model for a particular chemical problem at hand. While parsimony has traditionally been associated with training efficiency, new techniques show that, in some cases, more complex models can be easier to train and serve as faster alternatives to computationally demanding, parsimonious models.

#### 3.4.2. Usage.

Parsimony can enhance the resource efficiency of a model’s usage to the extent that a specific form of parsimony aligns with the tools we use to store and query models. If we measure parsimony via the number of components, and if computational or memory resources are dominated by terms related to the dimensionality of these components, then enforcing parsimony by components can help reduce computational requirements for storing and querying the model. For example, models using fewer variables typically need less memory to be stored and fewer computational operations (time and memory) to make predictions. For instance, using the recency heuristic which relies only on the latest data point to predict next week’s flu-related doctor visits is very resource efficient (123). Conversely, querying very large models with many components can be costly and slow (112). Therefore, parsimony often aligns well with the computational resources we use to store and query models.

However, models are often inefficient to use for reasons unrelated to parsimony, leading to instances where using more parsimonious models can be more costly than their complex counterparts. Recently, scientists have started developing complex models that emulate the functionality of inefficient parsimonious models. Although these emulators are expensive to train, they can be queried more efficiently than the parsimonious models they approximate (e.g., see refs. 124 and 125). Additionally, the structure of some complex models inherently enhances their efficiency. For example, complex yet sparse neural models can be efficiently queried by activating only a selectively small, parsimonious subnetwork for any given input. Similarly, gating mechanisms in large neural networks can mask or remove weights from certain subparts based on the context, making it efficient to query even very complex models. Thus, in some cases, parsimonious models can be less efficient to use than their more complex alternatives.

### 3.5. Suitability for Small and Noisy Datasets.

So far, we have discussed parsimony as a proxy for achieving certain model properties. However, parsimony has also been viewed as an unavoidable consequence of working with limited data. Some sciences almost always study phenomena that are only indirectly connected to empirical measurements. For instance, biological studies often aim to capture the multistep cascade process between the measured inputs and outputs. With each additional model component that is only loosely connected to empirical measurements, a model becomes harder to train, and the estimates of its parameters become less robust and less interpretable (126). Other limitations of data include the small number of observations or high amount of noise in them. For example, in psychological studies, people tend to differ in many unobserved and unmodeled factors, often only allowing the detection of high-level patterns, such as linear trends. This does not imply that relationships in data are inherently linear; rather, it reflects the fact that more intricate relationships might be indiscernible with the finite and qualitatively limited data at hand. The overarching theme in these examples is that parsimony in modeling can emerge as a consequence of acknowledging the boundaries of our knowledge and the restrictions of our data. Here, we suggest that parsimony is not a unique guide to models that are suitable for limited data.

Acknowledging the limitations of the data has traditionally pushed scientists to simplify their models, especially when carrying out more thorough experiments was not feasible. However, embracing overly parsimonious models in these situations is not the only possibility. As mentioned in Section 3.1.2, models that are more complex by constraints than needed to perfectly fit both the regularities and the noise in the data can achieve surprisingly high predictive accuracy. This means that extremely complex by constraints models can be used to extract reliable information from even a small number of observations. Moreover, acknowledging the fact that most causal processes that models aim to capture are not directly observable might motivate one to abandon the idea of converging on one “true” model with empirically unobservable components and to adopt a more pluralistic modeling approach (127–129). Here, the unobservable processes can instead be modeled by considering all the available supported accounts of the latent process resulting in a complex *ensemble* of models. For example, in climate science, forecasts are made by combining the predictions of multiple climate models proposed by different groups of scientists (22, 130). This approach integrates dozens of different structural models, each of which contributes a unique perspective on the Earth’s climate system, rather than requiring a conclusive selection of one account. Although such ensembles have so far been primarily employed for prediction, exploring the role they can play in improving scientific understanding of phenomena remains a promising direction for future research.

### 3.6. Alignment with Assumptions About Target Phenomena.

Scientists often use and interpret their models as reflections of the nature of their phenomena. If the phenomena themselves are believed to be simple, it could constitute an additional reason to use parsimonious models. For example, if epidemiologists believe that the dynamics relevant for virus transmission are self-contained (few parameters are needed to represent few relevant causal variables) and stable across contexts (few parameters are needed to account for little heterogeneity across contexts), this would motivate the use of parsimonious models to capture these phenomena. Whether constructing parsimonious models aligns with one’s beliefs about the nature of the target phenomenon varies between fields and individuals. Here, we suggest that the assumptions that inform the specification of parsimonious models do not always align with scientists’ beliefs about what they study.

Parsimonious-by-components models are more consistent with the idea that only a few components are necessary when describing target phenomena. The history of the natural sciences is full of examples justifying the use of more parsimonious models by referring to the nature of the world. For example, Copernicus defended the heliocentric model by appealing to a parsimonious world: “We thus follow Nature, who producing nothing in vain or superfluous, often prefers to endow one cause with many effects” (131). Similar arguments also appear in contemporary sciences. For instance, complex intelligent behavior is often thought of as guided by simple heuristics (132). Contemporary commitments to parsimony of phenomena are often implicitly reflected in how models are used and what inferences they license. For example, in experimental psychology and in medicine it is the norm to take the null hypothesis seriously (e.g., assuming that an untested drug has no effect), often because of the belief that a particular manipulation or treatment is more likely to result in no effect (133). In these ways, parsimony helps scientists to develop models that are better aligned with their beliefs about the nature of their phenomena.

Although parsimony sometimes aligns with discipline-specific assumptions about phenomena, there are cases where these assumptions are instead more consistent with more complex models. The spatial and temporal complexity of phenomena is being increasingly recognized across disciplines, including social and political sciences, psychology, neuroscience, biology, and physics. For example, psychological phenomena are commonly conceptualized as a result of a complex interaction of many contextual, often unobserved and unmodeled, factors (59). In such cases, a bias toward more parsimonious models, which are likely to omit these contextual variables, is misaligned with the appreciation of the importance of context in the discipline (58).

Viewing the target phenomena as either parsimonious or complex is not the only option. Some scientists believe that the true nature of the system of study is more complex, even if the data only allow them to discern simple patterns. For example, in psychology, data often reveal parsimonious trends, like linear relationships, even though most psychologists recognize that human psychology is far more intricate. Conversely, other scientists maintain that their system could be truly parsimonious, despite observations that appear complex (134). For instance, natural selection is often regarded as a parsimonious mechanism responsible for producing extraordinarily complex and diverse outcomes (135). Similarly, the premise of Chomskyan linguistics is that one can explain how the richness of human languages emerges through a parsimonious set of rules (2) (see ref. 136 for discussion of different levels of analysis). Thus, scientists often believe that parsimony and complexity may coexist in nature. Extending this idea to scientific methodology, we now turn to our conclusion on how parsimony and complexity can serve as complementary principles in science.

## 4. Conclusion: Complexity and Parsimony as Complementary Principles for Scientific Discovery

“*Less is more*”–*ancient Greek proverb*

“*The more the merrier*”–*English proverb*

In this paper, we discuss the relationship between parsimony and some desirable model characteristics. We articulate the circumstances under which parsimony could successfully lead a scientist toward models that are more predictively accurate, interpretable, useful in guiding future research, resource efficient, suitable for working with noisy and small datasets, and aligned with a field’s beliefs about the structure of their target phenomena. While parsimony often correlates with these desirable attributes of scientific models, this relationship is not universal and it depends on the specific interpretation of parsimony and on the modeling context (137). Models of different complexity instantiate different trade-offs, and so are best suited for different aims, contexts, and stages of scientific inquiry (138). Therefore, a universal preference for more parsimonious models can hinder the achievement of modeling goals. We suggest that preference for parsimonious and complex models can serve as complementary principles in the scientific process (88, 139).

Contemporary modeling practices increasingly call for a revision of model parsimony as a standalone property of a model. Rather, to serve as a useful proxy for achieving various modeling goals, parsimony might have to include the modeling context, such as training procedures and other elements of the modeling process. For example, while modern machine learning models might seem overwhelmingly complex in their number of free parameters, it is often the fitting procedures—such as gradient descent—that identify uniquely effective configurations of these parameters (60, 61). Thus, it might prove more useful to think about the entire modeling process, rather than just the model itself, as being parsimonious or complex (i.e., characterized by a certain number of constraints or components).

Parsimonious and complex models can be combined in scientific practice, often playing different roles at different stages of the scientific process. Traditionally, new ideas are introduced with models that are deliberately simple; application to data then leads to the discovery of new phenomena, which requires that the original models be adjusted and expanded in particular ways (13). One example of this is the gradual development of sequential sampling models for speeded response time tasks in cognitive psychology. The initial model (e.g., ref. 140) was relatively bare-bones, and it was gradually expanded by adding new processes (and associated parameters; e.g., ref. 141). Because the simple model could have been expanded in numerous ways, it would have been mere guesswork to propose any particular expansion before the availability of data to provide the proper guidance.

Recent advances reviewed in this paper point to the promise of an alternative strategy—namely, progressing from more complex to simpler models. Use of more complex models, particularly in initial stages of scientific exploration when prior knowledge is limited, can be instrumental in uncovering underlying structure in the data. Complex models with many flexible components afford more agnostic learning, reducing the risk of imposing incorrect assumptions on the data and instead learning as much as possible from the data itself. Once we have a successful complex model capturing the structure of the data, this model can be effectively compressed into a more parsimonious account for future use—for example, for gaining higher-level insight into the important aspects of the phenomena that were captured by the complex model. This can be achieved by such methods as *distilled learning* and *sparse approximators* (142, 143). These techniques demonstrate the feasibility of distilling trained complex models into more parsimonious, but equally accurate counterparts, while learning these parsimonious models directly from the data results in accounts that are less capable of capturing useful structure in the data (144–147). Here, it is learning a more complex model first that facilitates the discovery of a useful parsimonious model, presenting an exciting avenue for the multistage process of developing scientific models by starting with more complex models to learn from the data and then converting them into more parsimonious accounts for future use. In a recent application of this multistage modeling approach, psychologists used a complex machine learning model to fit the structure in the Moral Machine dataset comprising roughly 40 million moral decisions by human participants (148). This complex model was then used to refine more parsimonious and theoretically grounded models of moral judgment, leading to clarification of psychological dimensions that contribute to human moral decisions. This study presents a successful example of how starting with complex models to uncover rich structure in the data and then using them to inform more parsimonious accounts could enhance our understanding of the scientific phenomena of interest.

The appropriate role for parsimony in the modeling process depends not only on the modeler’s goals and context, but on science itself: Advances in statistics, computer science, cognitive science, and other fields continue to both refine and challenge our understanding of when, how, and in what ways parsimony facilitates or hinders scientific progress. Despite centuries of research since Ockham’s famous invocation of the principle of parsimony 700 y ago, this paper has highlighted that there remain many open questions and unexplored nuances of the principle of parsimony. We expect the principle of parsimony to both facilitate the evolution of and evolve alongside science itself.

## Data Availability

There are no data underlying this work.

## Footnotes

^*^The main goal of these methods is arguably to assess predictive accuracy; the importance of parsimony arises as an automatic by-product [e.g., note their connection to cross-validation (19, 20)].

^†^There are other ways to define parsimony—e.g., by directly associating it with desirable model properties such as interpretability or the ease with which the model can be developed and analyzed. In this paper, we do not adopt a definition of parsimony that takes any of these model properties for granted; instead, Section 3 explores whether parsimony, defined as either of the two forms above, serves as a successful proxy for the other desirable model properties.

^‡^A model can predict one or several of many different aspects of a target phenomenon, and so a potential data pattern can refer to many different types of outcomes. For example, different models make predictions at different levels of abstraction, ranging from very specific outcomes (e.g., it will rain tomorrow at 3PM in Moscow) to more temporally and spatially extended characteristics (e.g., the average amount of precipitation in Moscow across months will follow a normal curve with the peak in February).

^§^Interestingly, although these two understandings of parsimony are often used interchangeably, empirical studies frequently focus on one notion while using the general “parsimony” term. This distinction will be evident in Section 3, where some subsections emphasize one interpretation of parsimony—e.g., predictive accuracy focuses on parsimony by constraints while interpretability often focuses on parsimony by components.

^¶^The aim of this paper is to assess the relationships between model parsimony and these specific model properties; which modeling goals to prioritize is a key decision that remains at the discretion of readers and modelers.

^#^Human sense of understanding can diverge from actual understanding (71, 72). Even scientists have been shown to misinterpret very parsimonious models. For example, Soyer and Hogarth (2012) found that academic economists made faulty inferences when interpreting a simple linear regression with one variable, also failing to notice their lack of insight (73).

^‖^These cognitive constraints are mostly relevant to human-centric science; AI-augmented science may move past the human cognitive limitations constraining the ways models are used to guide new research.
